# Repeatedly heated mix vegetable oils-induced atherosclerosis and effects of *Murraya koenigii*

**DOI:** 10.1186/s12906-020-03012-4

**Published:** 2020-07-14

**Authors:** Gul Ambreen, Afshan Siddiq, Kashif Hussain, Abdul Saboor Hussain, Zara Naz

**Affiliations:** 1 Department of Pharmacology, Faculty of Pharmacy and Pharmaceutical Sciences, University of Karachi, Karachi, Pakistan; 2 Department of Pharmacy, Aga Khan University Hospital, Stadium Road (Main Pharmacy), P.O Box 3500, Karachi, 74800 Pakistan; 3 Institute of Pharmaceutical Sciences, Peoples University of Medical and Health Sciences, Nawabshah, Sindh Pakistan

**Keywords:** *Murraya koenigii*, Repeated heated, Vegetable oils, Oxidative stress

## Abstract

**Background:**

Statins are considered as standard drugs to control cholesterol levels, but their use is also associated with renal hypertrophy, hemorrhagic stroke, hepatomegaly, and myopathy. *Murraya koenigii* is an herb that is used in traditional cuisine and as a medicine in South Asia. Here we assessed the antidyslipidemic and antiatherosclerotic effects of this spice in repeated heated mix vegetable oils (RHMVO)-induced atherosclerotic models.

**Methods:**

Aqueous extract of *M. koenigii* leaves (Mk LE) was prepared and its phytoconstituents were determined. Rabbits were divided into 5 groups (*n* = 10). Except for the control group, all the other four groups were treated with RHMVO for 16 weeks (dose = 2 ml/kg/day) to induce dyslipidemia and atherosclerosis. These groups were further treated for 10 weeks either with 300 and 500 mg/kg/day Mk LE, lovastatin, RHMVO, or left untreated. Body and organ weights were measured along with oxidative stress and tissue damage parameters. Lipid profile and hepatic function markers were studied. Atheroma measurement and histopathological examination were also performed in control and treated groups.

**Results:**

Mk LE significantly (*p* < 0.05) attenuated RHMVO-induced dyslipidemia and atheroma formation. Furthermore, fat accumulation and lipid peroxidation in hepatic tissues were reduced by Mk LE in a dose-dependent manner. Our results indicated that the antidyslipidemic effects of Mk LE in 500 mg/kg/day dose were comparable to lovastatin. Additionally, oxidative stress markers were reduced much more significantly in Mk LE-500 than in the statin group (*p* < 0.05).

**Conclusions:**

This study recommends Mk LE as a potent antioxidant and lipid-lowering natural medicine that can attenuate the RHMVO-induced atherosclerotic in optimal doses and duration. Therefore, Mk LE can be accessible, cheap, and free of adverse effects alternate to statins.

## Background

In modern society, altered food consumption pattern increases incidences of metabolic syndrome, including obesity, hypertension, diabetes, atherosclerosis, stroke, and cancer [1–4]. Particularly dyslipidemia is considered as the basic cause of health concerns, related to metabolic syndrome [2]. As per National Cholesterol Education Program Adult Treatment Panel III (NCEP-ATP) guidelines, dyslipidemia is characterized as triglycerides (TG) > 150 mg/dl, total serum cholesterol (TC) > 200 mg/dl, low-density lipoproteins (LDL) > 100 mg/dl and high-density lipoproteins (HDL) less than 40 mg/dl. Dyslipidemia is the most important risk factors of cardiovascular diseases (CVDs) and atherosclerosis [5, 6].

Cholesterol is transported to the liver for metabolism, by HDL, whereas endothelial injuries and formation of atheroma are triggered by oxidized LDL molecule. Therefore, reduction of LDL and TC, along with HDL level enhancement is targeted to prevent atherosclerosis [7]. So far, statins have been utilized for inhibiting cholesterol biosynthesis, by restraining 3-hydroxy-3- methylglutary-coenzyme A (HMG-CoA). In addition, statins induce the cellular level of LDL receptors activity that in turn facilitates the metabolism of LDL and very-low-density lipoproteins (VLDL) [8, 9]. However, despite this benefit, long-term use, and/or over-dosage of statins results in serious adverse effects, such as renal hypertrophy, hemorrhagic stroke, hepatomegaly, and myopathy [10–12]. These facts are driving the researchers to explore the solution from nature to regulate blood flow and control the blood cholesterol levels, with the least adverse effects.

Physico-chemical characteristics of the cooking oils change when they are heated beyond a certain limit as several chemical reactions take place in the presence of moisture and air. Oils degenerate and produce volatile substances, unwanted monomers, polymers, isomers, and free radicals [13]. In cooking oil fatty acids (FAs) naturally exist in the cis-isomer form, but during thermal oxidation they convert into trans-isomers, possessing physical properties similar to saturated FAs [14]. Several animal studies demonstrate that consumption of thermally oxidized vegetable oils increased the risk of CVDs, like hypertension with reduced vasorelaxation responses [15], endothelial malfunction [16], lipid peroxidation [17], atherosclerosis [18], and oxidative stress [19]. However, regardless of all these studies, the practice of reusing and overheating the cooking oils while processing the food seems to continue.

*M. koenigii* (curry leaves) are commonly used spices in Pakistani cuisine. With the vast range of biological activities such as antidyslipidemic [20–22], platelet aggregation inhibiting [23], antioxidant [24], and hypoglycemic activities [25], it gained the attention of researchers worldwide. Several animal studies have reported this spice as a potent antidyslipidemic agent and the response was achieved in 4–8 weeks, in a dose-dependent manner [20, 21]. A recent human study, conducted on 45–65 years women of postmenopausal phase with hyperlipidemia, also reported that *M. koenigii* leaves intake for 45 consecutive days can significantly reduce the blood lipid levels [22]. A study also reported that Mk LE inhibits platelet aggregation, resulting in improved blood flow [23]. It also acts as an antioxidant, further suppressing the vascular inflammatory response through reduced reactive oxygen species (ROS) production [26].

Presently, several animal models have been used to study hyperlipidemia induced atherosclerosis, like mice [27], zebrafish [28], rats [29, 30], and pigs [31]. To study the effect of curry leaves on thermally oxidized oil-induced hypercholesterolemia and atherosclerosis selection of experimental animal models is very critical to conduct basic research and develop a study tool. In this respect, to study human hyperlipidemia the rabbit has become the most appropriate animal model because of rabbits’ unique features of lipid metabolism similar to humans [32, 33]. Several studies also used rabbit model for cardiac diseases [34]. Rabbit as an experimental animal is also considered appropriate for the Rabbit as an experimental animal model is also used to evaluate the medicinal effects of several herbal medicines, like the hypolipidemic effect of *Ficus bengalensis* [35], the hypoglycemic effect of *Achyranthes aspera* [36] antihyperglycemic effects of *Alpinia galanga rhizome* and its extracts [37], and hypoglycemic effects of *M. koenigii* [25].

In Pakistan, commercially available oils are mostly a blend of two or more edible oils, and the most common available blend of an equal ratio of olive, canola, and sunflower oils was focused in this study, to have the model that mimics the human situation. Vegetable oils are repeatedly heated when used for food processing (frying) at household and commercial levels. We used repeatedly heated mixed vegetable oils (RHMVO) to induce hyperlipidemia and atherosclerosis. We designed this study to investigate the medicinal effects of curry leaves in different doses, in RHMVO-induced atherosclerotic models.

## Methods

### Collection and identification of oils and plant material

Fresh curry leaves, standard food-grade canola, sunflower, and olive oils were purchased from Karachi local market. The specimen of plant material and oils were verified by the Department of Pharmacognosy, University of Karachi.

### Oil sample preparation

Olive, canola, and sunflower oils were mixed in equal ratio and heated beyond the smoke point of the oil mixture at 220 °C for 45 min each day for a total of ten days [38–40]. The smoke point for olive oil, canola oil, sunflower oil is 180 °C, 200 °C, and 225 °C, respectively [41, 42]. Every day before heating, the oil level was adjusted in the pan with the same oils blend (0.007 ± 0.003 l per day) to meet the initial level. This replenishment method was adopted to replicate the same practices used in fried and fast food outlets [38]. At the end of the 10th day, viscous dark brown oil was obtained. To prevent photodegradation it was stored in amber color bottles and labeled as RHMVO [43].

### Preparation of *Murraya koenigii* leaves extract (Mk LE)

Curry leaves were cleaned by thorough washing to remove any contamination with double-distilled water. Washed curry leaves were dried in shade at room temperature and grounded to powder with the help of a mechanical grinder and then preserved at room temperature in an airtight container. Three hundred grams powdered leaves were subjected to cold extraction by stirring them in 4-l double-distilled water for 24 h. The resultant suspension was centrifuged at 10,000×g for 10 min and filtered through Whatman No. I filter paper, followed by a 0.45 μm membrane filter (Sartorius minisart, Hannover, Germany). Finally, the filtrate was dried using a rotary evaporator and resulted in a 13.6% yield. The dried extract was dissolved in distilled water to achieve 100 mg/ml stock. Crude aqueous extract of curry leaves (Mk LE) was then kept at 4 °C storage temperature for subsequent use for planned experiments.

### Experimental animals

Healthy adult male rabbits of local strain weighing between 1450 ± 10 g were purchased from the department of pharmacology, University of Karachi, Pakistan. Male rabbits were selected to avoid the sex difference due to female sex hormonal effects on CVDs [44, 45]. Secondly, the prevalence of plasma lipid abnormalities in Pakistan, are higher in males than females [46, 47]. Rabbits were kept individually in wire-topped steel cages with a wooden bottom, under controlled humidity (50–60%), and temperature (23 °C ± °C) with 12/12 h light/dark photo-cycle. Animals were acclimatized for 7 days before starting the experiment. Animals were handled according to the institutional animal’s ethical committee guidelines.

### Experimental design

The total experimental period was of 26 weeks and all animals were divided into five groups (*n* = 10). In the first 16 weeks, except for the control, all other groups were treated with 2 ml/kg/day of RHMVO to induce hypercholesterolemia [43]. The induction period was followed by the treatment period of 10 weeks. In the treatment period animals were treated as follows

- Control group: Untreated
- RHMVO group: Rabbits continued to have 2 ml/kg/day RHMVO
- Mk LE-300 group: Rabbits were given Mk LE 300 mg/kg/day
- Mk LE-500 group: Rabbits were given Mk LE 500 mg/kg/day
- Statin group: Rabbits were given lovastatin 0.5 mg/kg/day

From the statin group, we selected lovastatin, to avoid the additional side effects (nasopharyngitis, diarrhea, and urinary tract infection) which are associated with other statins [48]. Oil and Mk LE were administered daily in the morning and lovastatin in the evening through the oral route, with the help of oral syringes [49]. Lovastatin, a short-acting statin, is prescribed to be taken in the evening to achieve better results as cholesterol production through livers enzymes is higher in these hours [50, 51]. For the rest of the day animals of all the groups were fed ad libitum on a regular normal diet (fresh hay and water). Morbidity and mortality were monitored through the study period. Animal’s body weight was recorded at baseline, week 1, 16, 20, 23, and 26. After the induction period (at 16 weeks) and treatment period (at 26 weeks), 3 rabbits from each group were euthanized through the intravenous administration of sodium pentobarbital 100 mg/kg followed by decapitation [52]. Furthermore, animals were necropsied for histopathological examination of internal organs and to retrieve liver, heart, spleen and kidney for organs weight evaluation.

### Blood sample collection for lipid profile and liver function markers

Blood samples were collected from the ear vein of animals at week 1, 16, 20, 23, and 26. For hematological parameters, 2 ml blood was collected in EDTA and 5 ml in gel vacutainers to perform the biochemical assays. Blood was centrifuged to collect plasma and serum and stored at − 20 °C until used further to analyze the plasma lipid profile (TG, TC, HDL, LDL, VLDL) and liver function markers, such as total protein, albumin, serum glutamic-pyruvic transaminase (SGPT), serum glutamic-oxaloacetic transaminase (SGOT) and alkaline phosphatase (ALP) using commercially available kits (RANDOX Laboratories Ltd) followed by manufacturer’s instructions.

### Oxidative stress and tissue damage parameters

Lipid peroxidation was measured by determining plasma malondialdehyde (MDA), following Kikugawa et al method [53]. The plasma C-reactive protein (CRP) was measured by using an ELISA kit (Abnova, Taipei, Taiwan), as per the manufacturer’s instruction. A commercially available kit was used to analyses lactate dehydrogenase (LDH) (BioVision, USA). We followed Hamsi et al. method to prepare the samples and standards [54]. Sheu et al. method was adopted to measures the fasting plasma homocysteine concentrations [55]. Creatine phosphokinase (CPK) was measured by a commercially available kit from Beckman Coulter, Brea, USA, following manufacturer instructions.

### Atheroma measurement

After 26 weeks animals were sacrificed as per the guidelines and necropsied to dissect a 10 cm aortic arch from the aortic valve and washed in normal saline followed by further dissection to open the orifice of the carotid artery (5 cm long) with a longitudinal cut. After removal of adhered tissue, propylene glycol (100%) was used to dehydrate the aorta, for 10 min followed by another 10 min staining 0.7% Sudan IV (in propylene glycol) [56–58]. The tissue was re-hydrated by treating it with 85% propylene glycol for 5 min, washed with distilled water, and photographed. Digital Image Analyzer was used to analyze the red atheromatous plaques. We measured the degree of lipid deposition by calculating the percent atherosclerosis index (AI) through the ratio between the Sudan-positive area to the whole aortic wall area.

### Microscopic examination

After weighing liver tissues, samples were treated with alcohol to dehydrate. After cleaning with xylene, samples were further embedded in paraffin at 56 °C. By using a rotatory microtome, 5 μm sized sections were obtained, mounted onto albumenized slides, and kept for drying for 12 h. Dewaxing was done in xylene and hydration was performed in ethanol and water. The sections were first treated with Harris hematoxylin to stain, thereafter differentiation by acid alcohol, and then staining in methylene blue was performed. Furthermore, dehydration of sections in alcohol (95%) and staining in alcoholic eosin (10%), dehydration with absolute alcohol, and cleaning with xylene were completed. The sections were then mounted using Canada balsam. These prepared slides were finally viewed with the use of a light microscope.

### Phytoconstituents determination of Mk LE

For the quantitative measurement of the total content of flavonoids and phenols in the Mk LE, we followed the methods of Sefi et al [59]. Total phenol content is mentioned in mg gallic acid equivalents (GAE) per each gram of extract, where GA was considered as standard. Total flavonoids are mentioned as mg catechin equivalents (CE) per each gram of extract, catechin was used as standard. For the quantitative finding of the total alkaloid content in the Mk LE, we followed the method of Sreevidya et al [60] and expressed as mg/g bismuth nitrate. Total tannin content was evaluated by following the Polshettiwar et al method [61] and expressed as tannic acid equivalent per gram of Mk LE. Total chlorophyll content was evaluated by following the method adopted in Kizhedath and Suneetha study [62] and expressed as mg pigments/gm.

### Statistical analysis

Data is represented in the form of mean ± standard deviation. We applied analysis of variance (ANOVA) followed by post hoc Tukey’s honest significant difference (HSD) test to find out statistical significance at *p* < 0.05. Statistical product and service solutions software was used.

## Results

### Biometric parameters

No death and unexpected signs and symptoms noticed in experimental animals throughout the study period in the treated animals and control. The body and organ weights of animals in all the groups increased gradually with age (Table 1 and Fig. 1). Bodyweight of all the animals treated with RHMVO was significantly higher than control, after the induction period of 16 weeks. At 26 weeks animals in the RHMVO group exhibited significantly higher weight than the age-matched other groups. However, statistically insignificant differences were present in the bodyweight of Mk LE-300, Mk LE-500 and lovastatin groups at 20th week, but at the end of 26th week, this phenomenon became very considerable and statistically significant body weight reduction was observed in Mk LE treated groups in a dose-dependent manner compared to RHMVO fed group (*p* < 0.05). Moreover, weight reduction was also statistically significant in the lovastatin treated group (*p* < 0.05), but lesser than the Mk LE-500 group. As shown in Table-1 organs weight reduction of Mk LE treated groups in the treatment phase was dose-dependent and statistically highly significant (*p* < 0.005) in 500 mg/kg/day doses. Organ weight reduction in Mk LE-500 treated group was even higher than the lovastatin treated group.

**Table 1:** Comparison of organs weight post-induction and after-treatment

Organs	Observation Time	Control (weight-g)	RHMVO (weight-g)	Mk LE-300 (weight-g)	Mk LE-500 (weight-g)	Lovastatin (weight-g)
Liver	Post induction (week16)	53.66 ± 1.35	86.25 ± 2.62	87.38 ± 1.88	85.26 ± 1.92	86.24 ± 2.22
	After treatment (week26)	60.31 ± 4.33	104.95 ± 2.6 **##**	83.76 ± 1.89	63.17 ± 2.56 **	75.51 ± 2.26*
Spleen	Post induction (week16)	34.37 ± 1.47	56.89 ± 2.15	57.1 ± 2.25	55.75 ± 3.09	55.09 ± 3.46
	After treatment (week26)	40.88 ± 3.42	74.96 ± 4.5 **#**	54.08 ± 2.26	42.48 ± 1.2**	51.27 ± 2.52
Heart	Post induction (week16)	18.08 ± 1.39	34.75 ± 0.9	34.92 ± 1.09	34.9 ± 1.14	34.95 ± 1.15
	After treatment (week26)	18.54 ± 5.92	49.17 ± 4.84 **#**	32.83 ± 1.09	29.31 ± 1.14*	31.86 ± 1.12
Kidney	Post induction (week16)	14.41 ± 0.9	27.74 ± 1.53	27.97 ± 1.63	27.77 ± 1.74	28.11 ± 1.94
	After treatment (week26)	17.98 ± 2.43	37.16 ± 2.18 **##**	25.99 ± 1.28	21.05 ± 1.38*	25.6 ± 0.55

*Significantly reduced from post-induction weight (*p* < 0.05)

**Significantly reduced from post-induction weight (*p* < 0.005)

# significantly increased from the post-induction weight (p < 0.05)

## Significantly increased from post-induction weight (p < 0.005)

**Fig. 1:**
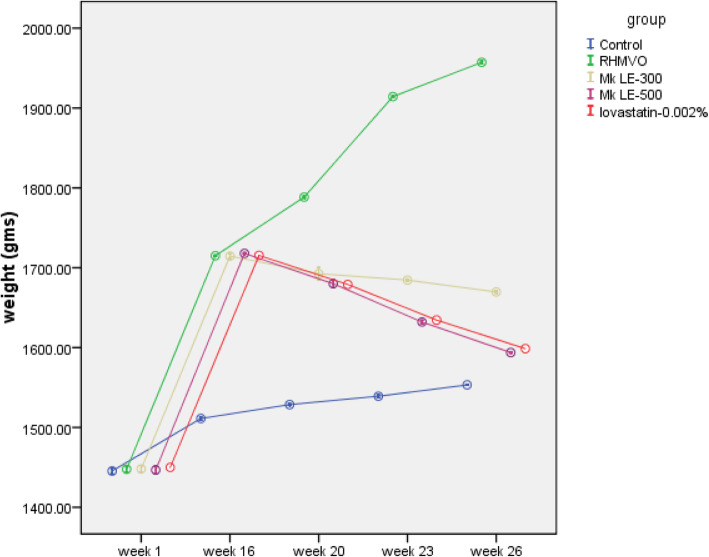
Graphical Presentation, Comparing the Weight Gain in Different Groups During the Study Period (at 95% CI): (**Control = Blue** line showing the gradual increment in bodyweight with age, **RHMVO fed group = Green** line showing significant weight gain, **Mk LE-300 group = Brown** line showing the weight reduction over week 16 to 26, **lovastatin group = Red** line showing significant weight reduction, **Mk LE-500 group = Purple** line showing the significant weight reduction comparable with red line of lovastatin group

### Serum biochemical analysis of lipid profile

As shown in Table 2, animals in RHMVO exhibited higher TGs, TC, LDL, VLDL, and lower HDL circulating levels in comparison with control. After 26 weeks of RHMVO feeding the TGs and TC values increased about 4–5 folds in the RHMVO group. Whereas in Mk LE treated groups all these parameters improved in a dose-dependent manner and statistically significant (*p >* 0.05) changes were observed in Mk LE-500 and lovastatin groups. Although, at weeks 20 and 23 there was no such obvious difference between Mk LE-300, Mk LE-500, and statin groups. But at the end of 26 weeks Mk LE-500 group animals presented significantly (*p* > 0.005) improved lipid profile than Mk LE-300 and comparable to statin group. At the end of treatment HDL levels were significantly higher in the Mk LE-500 group compared to the control group.

**Table 2:** Comparison of lipid profile through the study period (baseline till week-26)

Observation Time	Control	RHMVO	Mk LE-300	Mk LE-500	Lovastatin
**TRIG (mg/dl)**					
week 1-baseline **(Before induction)**	56.32 ± 3.51	55.43 ± 2.21	55.62 ± 3.28	57.08 ± 3.80	56.58 ± 3.18
week 16 **(Post induction)**	69.08 ± 2.92	272.18 ± 1.76**	279.61 ± 6.76**	281.4 ± 6.03**	276.01 ± 12.31**
week 20	73.17 ± 2.11	309.26 ± 1.83**	251.83 ± 6.16	256.01 ± 6.19	255.67 ± 12.05
week 23	74.75 ± 3.42	330.61 ± 1.55**	220.05 ± 6.28	218.76 ± 5.05	224.46 ± 12.92
week 26	75.93 ± 4.0	352.07 ± 6.74**	179.06 ± 6.09 **#**	166.51 ± 3.08 **##**	195.2 ± 12.41**#**
**TC (mg/dl)**					
week 1- baseline **(Before induction)**	141.02 ± 3.21	140.12 ± 3.25	141.21 ± 5.02	141.30 ± 3.55	141.25 ± 1.58
week 16 **(Post induction)**	148.54 ± 4.25	444.5 ± 8.23**	446.31 ± 8.59**	450.35 ± 8.91**	443.29 ± 11.61**
week 20	150.19 ± 5.23	503.45 ± 8.09**	394.07 ± 8.23	378.1 ± 8.09	388.08 ± 11.06
week 23	151.84 ± 3.02	562.4 ± 8.55**	350.75 ± 7.99	289.85 ± 8.23**#**	333.82 ± 11.31
week 26	154.4 ± 3.87	637.75 ± 9.99**	308.51 ± 8.32**#**	200.59 ± 8.59**##**	280.61 ± 11.38**##**
**LDL (mg/dl)**					
week 1- baseline **(Before induction)**	48.23 ± 3.29	47.22 ± 1.29	48.01 ± 3.31	47.59 ± 3.98	47.29 ± 3.19
week 16 **(Post induction)**	67.66 ± 3.31	367.61 ± 15.22**	366.21 ± 18.30**	363.57 ± 14.69**	361.63 ± 14.17**
week 20	68.87 ± 3.06	405.59 ± 18.23**	351.9 ± 15.09	326.36 ± 14.09	326.62 ± 14.29
week 23	69.72 ± 3.25	439.84 ± 10.59**	333.59 ± 11.66	271.24 ± 11.05	278.36 ± 10.09
week 26	73.92 ± 2.88	481.22 ± 75.05**	311.35 ± 18.36	213.59 ± 10.29##	240.65 ± 12.07#
**VLDL (mg/dl)**					
week 1- baseline **(Before induction)**	11.08 ± 0.56	11.22 ± 0.77	11.21 ± 0.81	11.10 ± 0.67	11.02 ± 0.66
week 16 **(Post induction)**	14.88 ± 0.74	82.18 ± 3.97**	82.73 ± 4.49**	84.27 ± 4.29**	82.55 ± 3.89**
week 20	15.84 ± 0.70	90.43 ± 3.29**	77.52 ± 4.20	73.06 ± 5.20	72.38 ± 3.34
week 23	16.65 ± 0.62	98.68 ± 5.02**	70.31 ± 4.35	55.85 ± 4.35#	60.49 ± 3.09
week 26	16.52 ± 0.53	105.55 ± 3.07**	62.2 ± 2.21	30.64 ± 4.04##	48.51 ± 4.52#
**HDL (mg/dl)**					
week 1- baseline **(Before induction)**	53.23 ± 8.69	53.33 ± 7.05	53.53 ± 6.05	54.59 ± 6.29	53.09 ± 8.34
week 16 **(Post induction)**	69.64 ± 3.35	16.08 ± 1.67$$	15.78 ± 2.17$$	15.03 ± 2.22$$	15.03 ± 2.19$$
week 20	70.92 ± 6.09	13 ± 1.38$$	21.99 ± 2.01	32.18 ± 2.56^	24.28 ± 2.65
week 23	71.18 ± 2.95	9.32 ± 2.08$$	40.08 ± 3.55^	67.43 ± 4.05**^^**	34.63 ± 5.29**^**
week 26	73.92 ± 5.01	5.64 ± 2.59$$	58.06 ± 3.23**^**	81.68 ± 2.92**^^ %**	42.89 ± 4.84**^**

**highly significant difference (p < 0.005) (increased values) than week 1- baseline (Before induction) values

**#** significant difference (p < 0.05) (decreased values) than week 16- (post induction) values

**##** highly significant difference (p < 0.005) (decreased values) than week 16- (Post induction) values

$$ highly significant difference (p < 0.005) (decreased values) than week 1- baseline (Before induction) values

**^** significant difference (p < 0.05) (increased values) than week 16- (post induction) values

**^^** highly significant difference (p < 0.005) (increased values) than week 16- (post induction) values

% significant difference (p < 0.05) (increased values) than control group in corresponding weeks

*TRIG* Triglycerides, *TC* Total cholesterol, *LDL* Low-density lipoproteins, *VLDL* Very-low-density lipoproteins, *HDL* High-density lipoproteins

### Hepatic function and oxidative stress markers

The biochemical analysis of liver function markers has shown that continuous feeding of RHMVO induced hypercholesterolemia, which resulted in significantly (*p* < 0.005) reduced total protein and albumin levels. The blood levels of LDH, CPK, SGOT, SGPT, and ALP were also increased significantly, suggestive of liver dysfunction with reduced protein synthesis and hepatic injury. With the treatment of Mk LE, hepatic function markers were restored in a dose-dependent manner, but significantly in Mk LE-500 group (*p* < 0.005). In lovastatin (0.5 mg/kg/day) treated group few parameters improved significantly after 10 weeks of treatment (*p* < 0.05).

At the end of the induction period, oxidative stress and inflammation were also significantly (*p* < 0.005) increased with RHMVO feeding, shown by elevated MDA and CRP levels. Mk LE in higher doses of 500 mg/kg was enough to restore the normal levels after 10 weeks of treatment (*p* < 0.005). Lovastatin (0.5 mg/kg/day) treated group exhibited a lesser impact on inflammatory and oxidative stress markers over 10 weeks period (Table 3).

**Table 3:** Comparison of oxidative stress and liver function markers at week-26

Variables	Control ##	RHMVO**	Mk LE-300	Mk LE-500	Lovastatin
CPK (U/L)	148.17 ± 2.29	1116.85 ± 9.27	981.81 ± 8.55**	562.84 ± 13.78 ** ##	947.84 ± 17.41** #
LDH (U/L)	212.44 ± 45.38	1214.21 ± 9.71	986.64 ± 4.86** #	508.13 ± 9.05 * ##	831.42 ± 12.85 ** #
Homocysteine (μMol/L)	4.62 ± 0.54	15.88 ± 1.08	10.57 ± 1.33* #	5.26 ± 1.04 ##	11.33 ± 0.91**
MDA (nMol/ml)	8 .00 ± 1.43	29.7 ± 1.32	20.68 ± 2.42** #	12.14 ± 1.69 * ##	19.77 ± 1.71 ** #
CRP (mg/dl)	0.04 ± 0.01	14.64 ± 2.16	11.93 ± 1.52** #	4.39 ± 1.57 * ##	9.81 ± 0.98**
SGPT (U/L)	92.9 ± 4.65	376.23 ± 8.33	305.5 ± 2.85**	208.93 ± 70.98 * ##	301.82 ± 10.57**
SGOT (U/L)	95.28 ± 5.11	404.28 ± 8.77	311.41 ± 3.91**	185.3 ± 35.78 * ##	315.42 ± 6.45**
ALP (U/L)	82.89 ± 4.8	635.25 ± 4.98	556.35 ± 3.07**	217.2 ± 9.26 * ##	355.59 ± 6.82** #
Total Protein (mg/dl)	7.42 ± 0.43	2.5 ± 0.24	3.51 ± 0.25*	5.45 ± 0.29 * #	2.11 ± 0.13**
Albumin (mg/dl)	3.42 ± 0.46	1.08 ± 0.04	2.06 ± 0.05#	3.09 ± 0.12##	1.56 ± 0.43*

*Significantly different from Control (*p* < 0.05). #significantly different from RHMVO (*p* < 0.05). **Significantly different from Control (*p* < 0.005). ##significantly different from RHMVO (*p* < 0.005)

### Chemical analysis of the extract

Dry powder of curry leaves yielded 13.6% components by weight (water-soluble). The quantitative characterization of the chemical in Mk LE showed the significant existence of alkaloid, flavonoid, chlorophyll, polyphenol, and tannin (Table 4).

**Table 4:** Quantitative extraction yield and chemical content of Mk LE

Sample	Extraction yields	Chemical composition				
	(%) = weight of sample extract /sample weight) × 100	Total phenols (mg gallic acid equivalent /g Mk LE)	Total flavonoid (mg catechin equivalent. /g Mk LE)	Chlorophyll content (mg/g Mk LE	Alkaloid content (mg bismuth nitrate/g Mk LE)	Total tannin (mg tannic acid equivalent /g Mk LE)
*M.koenigii* leaf aqueous extract (Mk LE)	15.63 ± 0.46	46.78 ± 0.07	6.1 ± 0.36	0.40 ± 0.009	37.9 ± 0.28	0.129 ± 0.008

Values represented as means ± SD

### Effect on atheromatous plaques area

**Fig. 2:**
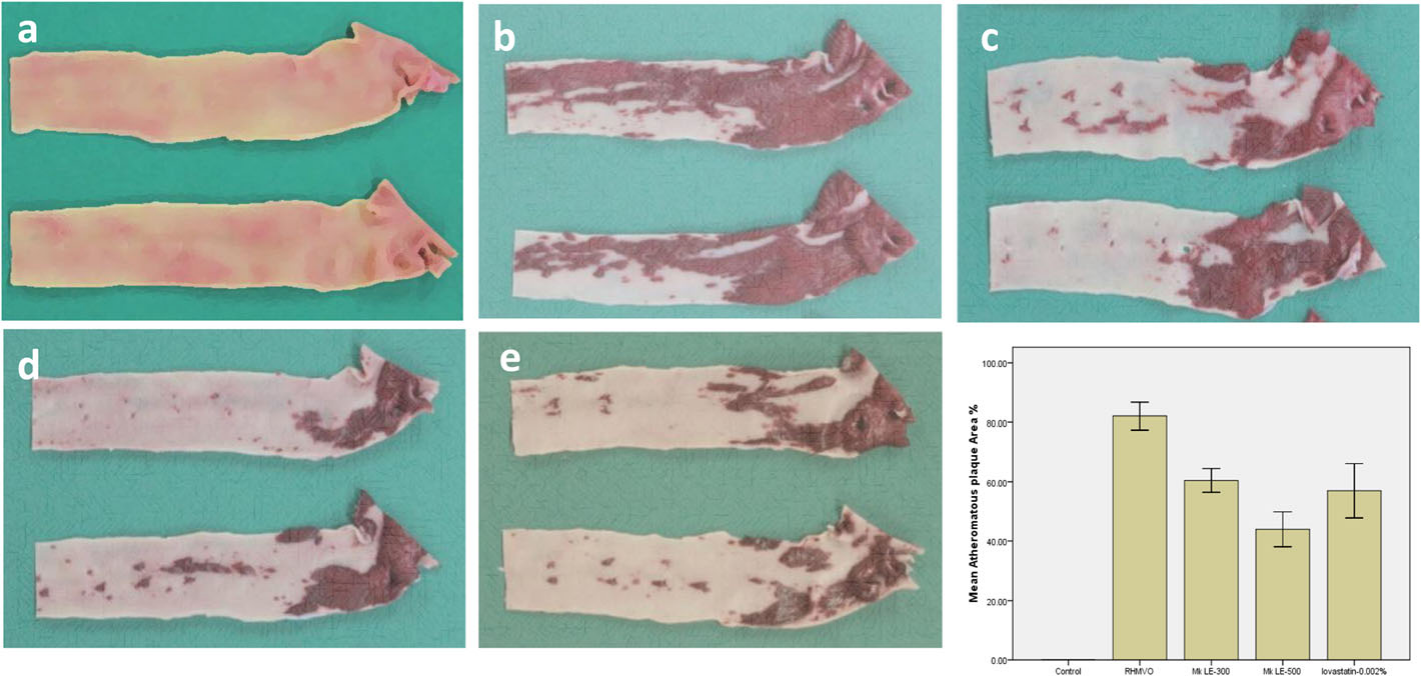
Atheroma formation and resolution at the end of 26 weeks: (**a**) **Control**; with no atheromatous plaques formation (**b**) **RHMVO** fed group; extensive atheromatous plaques formation, covered about 80–85% of the aortic arch (**c**) **Mk LE-300** group; atheromatous plaque reduced to 60% (**d**) **Mk LE-500** group; atheromatous plaque reduced to 40% (**e**) **lovastatin group**; Atheromatous plaque reduced to 57%

As shown in Fig. 2, at the end of 26 weeks RHMVO feeding resulted in extensive atheromatous plaques formation, covered about 80–85% of the aortic arch. This atheromatous plaque was noticeably attenuated by Mk LE in a dose-dependent manner at the end of the treatment phase. Atheromatous plaque reduced to 60 and 40% in 300 mg and 500 mg Mk LE doses respectively. The atheroma area was reduced by lovastatin (0.5 mg/kg/day) to 57% after 10 weeks of treatment.

### Hepatic histological observations

**Fig. 3:**
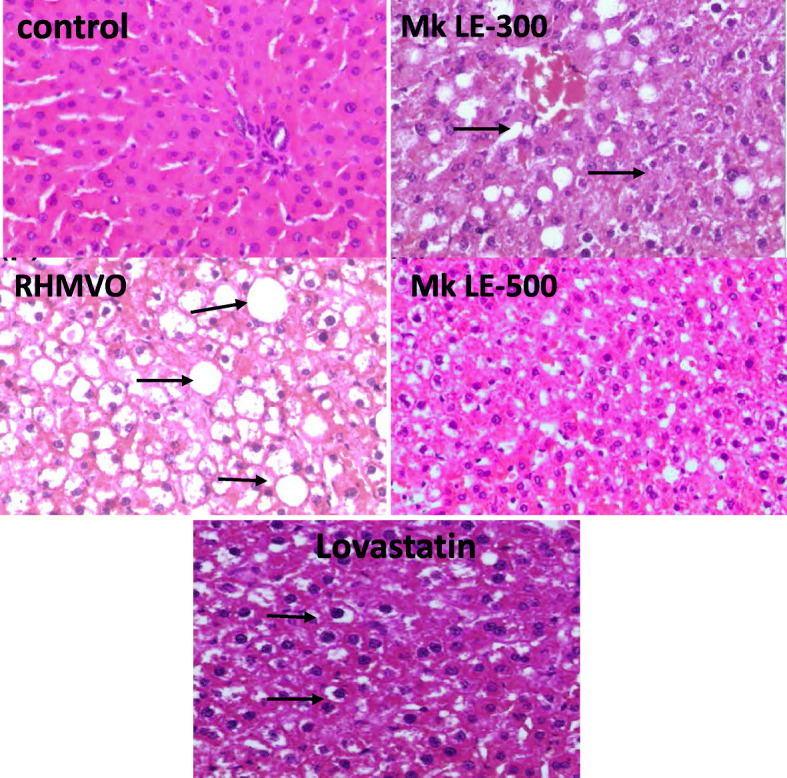
Histopathological finding of rabbits’ liver after 10 weeks of treatment**. Control**: a microscopic view of a normal liver. **RHMVO** fed group: a microscopic view of fatty liver showing distended hepatocytes due to fat vacuoles. **Mk LE-300** group: observed a lesser number of fat vacuoles in the portal area. **Mk LE-500** group: observed an insignificant number of fat vacuoles. **Lovastatin** group: observed an insignificant number of fat vacuoles (200x magnification)

Under the microscopic observations, widespread lipid accumulation was observed in hepatocytes in the RHMVO group (Fig. 3). The point of interest is the remarkable dose-dependent attenuation of RHMVO-induced hepatic steatosis with Mk LE treatment. The effect of Mk LE-500 is greater than the lovastatin.

## Discussion

Alternative medicines are getting an important place to manage different health problems and rising as a wide field of research. Management of hyperlipidemia-induced atherosclerosis also relies on alternative medicine as an upcoming changed trend to protect and treat. Using the animal model, we created a real-life scenario and induced hyperlipidemia and atheroma formation with RHMVO and considered herbal nutritional sources to manage it.

The results of this study establish that Mk LE has the potential to treat the RHMVO-induced hyperlipidemia and atherosclerosis. The extract is found as a rich source of polyphenols, alkaloids, chlorophyll, and flavonoids. The quantitative analysis of curry leaves aqueous extract reported that there are constituents with antioxidant properties, the main reason for using curry leaves as alternative medicines and nutritional supplements in oxidative stress-induced disease studies [63–65]. Mk LE, with its immense health benefits, has been reported as a protective remedy against oxidative stress-induced diabetes [66]. The aqueous extract of curry leaves also has prolific antioxidant activities against lead and cadmium-induced oxidative stress in animal models [66, 67]. Several in vivo [68, 69] and in vitro [70, 71] studies reported the free-radical scavenging ability and antioxidant potential of curry leaves extract concluding the immediate and strong ameliorative actions for treating oxidative stress. Considering the same facts, in this study we evaluated the dose-dependent effect of curry leaves, to treat the hyperlipidemia and oxidative stress induced by the high dose of thermally oxidized cooking oils blend. The impact was also compared with standard i.e. lovastatin from the statin group. Mk LE at a dose of 500 mg/kg was found effective in a shorter period, to treat oxidative damage and hyperlipidemia mediated by RHMVO compared to 300 mg/kg. The histopathological and macroscopic results showed that to treat RHMVO-induced atherosclerosis the effect of MK LE in 500 mg/kg of body weight is comparable with lovastatin, as all the study parameters were significantly reduced in the Mk LE-500 group.

Thermally oxidized oil is the most significant source of oxidative damage for human health if used daily for a long time. In RHMVO fed groups, highly elevated levels of SGOT (extremely sensitive and a precise biomarker for hepatotoxicity) and ALP (a main biomarker of hepatic and biliary defects like cholestasis) [72] reflected hepatocytes damage and tissue interruption, allowing the leakage of intracellular enzymes in blood [73]. The liver is damaged by excessive free radicals and may result in hepatitis, cirrhosis, and hepatic tumor [74]. Consistent with the diminished serum albumin and total protein levels and supported by the significantly enlarged liver and histopathological changes in hepatocytes of RHMVO fed rabbits. The results of our study are consistent with the previous study, reported significantly reduced serum SGPT and SGOT levels in curry leaves pretreated groups against the lead-induced model, a clear indication of hepatic protection [75, 76], but the maximum effective dose of curry leaves was 100 mg/kg body weight.

The noticeable presence of carbazole alkaloids and tannins in Mk LE analysis exhibit excellent hepatoprotective activity through their anti-lipid peroxidation and antioxidant potential [77]. In the present study, Mk LE considerably inhibited fat accumulation in hepatocytes and the formation of hypercholesterolemia-induced atheroma. Besides, curry leaves extract attenuated lipid peroxidation and oxidative membrane injury in the organs showing lipidosis. Statins are preferentially prescribed to improve blood lipid profile through suppressing hepatic cholesterol synthesis [11]. However, it’s reported in previous studies that long-term use and over-dosage of the statins results in severe hepatotoxicity, whereas low doses failed to control blood cholesterol levels effectively from the diet [10–12]. Statins are reported to produce adverse effects in up to 33% of patients [78]. Notably, the management of cerebrovascular and cardiovascular disease risk factors in preventive mode is more effective than the therapeutic mode, as after the outbreak of these diseases time to death is very short [11]. Therefore, it has been recommended to follow dietary restrictions and choose appropriate oils and fats. In addition, the excessive consumption of oils and fats especially in thermally oxidized form is also known to cause vascular diseases, such as reduced vasorelaxation responses, endothelial malfunction [16], lipid peroxidation [17], atherosclerosis, and oxidative stress [19].

Reactive oxygen species (ROS) are free radicals with one or more unpaired electrons. Major ROS include superoxide anion, hydrogen peroxide, and the hydroxyl radical [79]. In addition to ROS, reactive nitrogen species (RNS), including peroxynitrite (NO3-), nitric oxide radical (NO), and S-nitrosothiols, also contribute to the generation of oxidative stress. ROS are often generated as byproducts of cellular metabolic reactions and exogenous induction. These ROS produce homeostatic imbalances, and overwhelm cellular antioxidants defense system and results in oxidative stress, that further damage biomolecules such as proteins, nucleic acids, and lipids which eventually, induces cell death and tissue injury [80, 81]. Subsequently promotes the development of age-dependent diseases, like cancer, atherosclerosis, arthritis, etc. [82]. MK LE has several potent antioxidants, such as isomahanine, isolongifolene, mahanine, mahanimbine, koenimbine,girinimbine, koenoline, and O-methylmurrayamine [83, 84]. These antioxidants exhibit 2,2-diphenyl-1-picrylhydrazyl (DPPH) free radical scavenging activity, inhibition of NO radical, and thiobarbituric acid reactive substances (TBARS) activity [85, 86].

Mitochondria are the powerhouse of the living cell and play a vital role in scavenging free radicals and controlling programmed cell death and/or the apoptosis-signaling pathway [87]. RHMVO-induced mitochondrial damage results in decreased adenosine triphosphate (ATP) production, increased ROS generation, impaired calcium buffering, damage to mitochondrial DNA (mtDNA). An altered mitochondrial morphology and alterations in mitochondrial fission and fusion. Mitochondrial dysfunction is characterized by a loss of efficiency in the electron transport chain, as well as reductions in the synthesis of high-energy molecules [88]. Mitochondrial dysfunction is a characteristic of all chronic diseases including neurodegenerative disorders, CVDs, diabetes, auto-immune diseases, atherosclerosis, and others [89, 90].

Recent studies have evaluated the neuroprotective activities of compounds isolated from MK LE. A study reported that the bioactive compounds present in MK LE exhibited the ability to repair the mitochondrial damage and restore the mitochondrial membrane potential levels [91–93]. Previous findings have demonstrated that MK LE and its primary active compounds regulate multiple signaling pathways, including phosphatidylinositol 3 kinase (PI3K)/protein kinase B (AKT), mammalian target of rapamycin (mTOR) and mitogen-activated protein kinase (MAPK). *M. koenigii* and its primary active compounds exert complementary effects on oxidative stress and the alteration of proteins [94, 95].

The lipid-lowering activity of MK LE is reported in several animal studies, in the different doses, in the range of 80–600 mg [20, 21, 23]. To our knowledge, this is the first animal study that evaluated the effect of MK LE, in RHMVO-induced hypercholesterolemia in animals, also using the standard (lovastatin). In the dose of 500 mg/kg MK LE significantly reduced the TG, total cholesterol, LDL, and VLDL, also, increased HDL levels and reduced the cardiac infarction risk through the recovery of the arterial endothelial cells functions [96]. Better response with high doses is also supported by other studies [20, 97]. The requirement of a longer duration to treat dyslipidemia is also supported by a previous study [97].

The high value of CRP is possibly indicative of developing cardiovascular disease, active inflammation, bacterial infection [98], and inflammatory bowel disease, and other similar conditions like intestinal lymphoma and tuberculosis [99]. Overexpression of CPK, homocysteine, and LDH has been linked with the number of disorders including tissue damages, ROS induced inflammation, and endothelial cell injury [100]. The elevated levels of all these tissues damaging markers are suggestive of increased risk of hypertension, diabetes, and CVDs [101, 102]. Reduction in these parameters along with reduced oxidative stress is an additional benefit of MK LE, compared to statins.

Moreover, it was demonstrated that MK LE inhibits platelet aggregation when given with coriander extract [23], thus further explains the MK LE potential to treat RHMVO-induced atherosclerosis. Formation of thrombus and atherosclerosis takes place when vascular endothelial walls get injured during oxidized LDL-mediated oxidative reactions and activated platelets get attached to this area. Therefore, curry leaves extract can be the alternate option for investigators to treat hyperlipidemia, oxidative stress, and atherosclerosis medicated by consumption of a higher amount of RHMVO in daily life.

## Conclusion

The results of this study conclude that Mk LE exhibits potent antioxidant and antidyslipidemic properties that can be used to treat RHMVO-induced hyperlipidemia and atherosclerosis in optimal doses and duration. Over the past few decades, the prevalence of coronary artery diseases has increased significantly in South Asians and predicted to be more than 24 million in 2035. Curry leaves are basically of south Asian origin, so it can be the better, easier, and cheaper option as nutrient supplement and remedy.

## Data Availability

The datasets used and/or analyzed during the current study are available from the corresponding author on reasonable request.

## Abbreviations

LDL: Low-density lipoproteins
VLDL: Very-low-density lipoproteins
TG: Triglycerides
TC: Total cholesterol
HDL: High-density lipoproteins
CVDs: Cardiovascular diseases
HMG-CoA: 3-hydroxy-3- methylglutanyl-coenzyme A
ROS: Reactive oxygen species
MDA: Malondialdehyde
CPK: Creatine phosphokinase
LDH: Lactate dehydrogenase
CRP: C-reactive protein
